# Mitochondrial Transporter ABCB10 Protects Against Doxorubicin‐Induced Respiratory Muscle Dysfunction Independent of Changes to Diaphragm Accumulation

**DOI:** 10.1002/jcsm.70171

**Published:** 2026-01-16

**Authors:** Ashley J. Smuder, Vivian Doerr, Cesar E. Jacintho Moritz, Jie Li, Branden L. Nguyen

**Affiliations:** ^1^ Department of Applied Physiology and Kinesiology University of Florida Gainesville Florida USA

**Keywords:** ATP‐binding cassette transporter, heme, iron, multidrug resistance, reactive oxygen species

## Abstract

**Background:**

Doxorubicin (DOX) is a highly effective chemotherapeutic agent whose use can cause respiratory toxicity, increasing patient fatigue and negatively impacting quality of life and survival. These adverse effects occur due to diaphragm muscle mitochondrial accumulation of DOX, where it causes reactive oxygen species production and iron dysregulation. ABCB10 is a mitochondria‐localized ATP‐binding cassette transporter hypothesized to play a role in the maintenance of mitochondrial redox balance and iron homeostasis, and potentially the mitochondrial export of DOX. This study investigated potential therapeutic effects of ABCB10 to prevent DOX‐induced respiratory muscle dysfunction.

**Methods:**

DOX respiratory muscle toxicity was modelled in rats using both single (20 mg/kg, once) and multicycle (5.7 mg/kg, 3 cycles) administration. The effects of overexpression and knockdown of ABCB10 on DOX‐induced diaphragm dysfunction, mitochondrial DOX accumulation and markers of mitochondrial iron homeostasis were evaluated via administration of rAAV9‐MHCK7‐ABCB10 or an antisense oligonucleotide targeting ABCB10, respectively.

**Results:**

ABCB10 significantly improved diaphragm rate of fatigue (138.2 ± 11.66 s vs. 104.6 ± 8.79 s in DOX), specific force production (22.12 ± 0.70 N/cm^2^ vs. 18.31 ± 1.65 N/cm^2^ at 160 Hz in DOX) and fibre area (Type I: 1309.05 ± 56.86 μm^2^ vs. 1027.04 ± 50.53 μm^2^ in DOX; Type IIa: 1389.13 ± 47.72 μm^2^ vs. 1027.04 ± 50.07 μm^2^ in DOX; Type IIb/x: 2590.81 ± 103.21 μm^2^ vs. 2302.13 ± 138.62 μm^2^ in DOX) following a single injection of DOX. These improvements did not occur as a result of ABCB10‐induced efflux of DOX (1006.03 ± 214.30 pg/μg vs. 1008.69 ± 195.62 pg/μg in DOX) but were associated with reduced mitochondrial iron (0.17 ± 0.02 nmol/mg vs. 0.23 ± 0.02 nmol/mg in DOX). The beneficial effects on diaphragm rate of fatigue (136.5 ± 11.93 s vs. 121.5 ± 9.47 s in DOX), specific force production (23.36 ± 1.40 N/cm^2^ vs. 19.26 ± 1.21 N/cm^2^ at 160 Hz in DOX) and fibre area (Type I: 1124.68 ± 63.02 μm^2^ vs. 914.57 ± 63.09 μm^2^ in DOX; Type IIa: 1244.67 ± 106.18 μm^2^ vs. 950.02 ± 62.38 μm^2^ in DOX; Type IIb/x: 2548.37 ± 235.69 μm^2^ vs. 2222.17 ± 234.61 μm^2^ in DOX) were also present in rats that received multiple cycles of DOX. Diaphragm rescue with ABCB10 was attendant with reduced mitoferrin 1 gene expression (+1.62 ± 0.47 fold vs. +3.85 ± 0.99 fold in DOX), preservation of mitochondrial function and a reduction in markers of heme synthesis, including Fech (+0.78 ± 0.17 fold vs. +0.24 ± 0.09 fold in DOX), ALAS1 (+0.54 ± 0.18 fold vs. +0.19 ± 0.04 fold in DOX), ALAS2 (+0.68 ± 0.23 fold vs. +0.33 ± 0.11 fold in DOX) and CPOX (+0.84 ± 0.16 fold vs. +0.38 ± 0.09 fold in DOX). While increasing ABCB10 in the diaphragm prevented DOX respiratory toxicity, reducing its expression did not exacerbate diaphragm dysfunction or mitochondrial DOX and iron accumulation.

**Conclusions:**

These results suggest that ABCB10 can preserve mitochondrial and diaphragm muscle function following DOX treatment by regulating iron redox‐cycling and heme synthesis, independent of changes to DOX accumulation.

## 1 Introduction

Doxorubicin (DOX) is a highly effective antineoplastic agent used in the treatment of hematologic and solid tumour cancers. The potent anti‐tumour activity of DOX is attributed to a wide spectrum of actions including DNA intercalation, topoisomerase II inhibition and reactive oxygen species (ROS) generation [1]. However, the pleiotropic effects of DOX are not limited to cancer cells, and clinical use is limited due to off‐target cellular toxicity resulting from nonselective tissue distribution and uptake [2].

In muscle tissue, DOX preferentially accumulates within the mitochondria, where it localizes to the matrix and impairs mitochondrial function through redox cycling, increases free radical generation and induces cell death, leading to muscle weakness [2, 3]. The diaphragm muscle is particularly susceptible to DOX toxicity, with respiratory muscle dysfunction a primary contributor to patient reported dyspnea, fatigue and exercise intolerance [4, 5, 6]. These consequences of reduced respiratory muscle strength following DOX treatment strongly affect quality of life and are predictors of poor survival among cancer patients [7, 8]. In fact, breast cancer survivors at least 3 months removed from treatment showed an ~20% reduction in respiratory muscle strength and greater chronic activity‐related dyspnea compared to healthy age‐matched control subjects, and additional clinical reports linked perceived dyspnea to diaphragm muscle weakness caused by DOX treatment [4, 5, 6].

While increasing evidence in clinical and preclinical studies demonstrates increased diaphragm muscle fatigue properties and decreased force production following DOX treatment, the precise mechanisms driving diaphragm mitochondrial accumulation of DOX and subsequent dysfunction remain unclear [3, 6, 9, 10]. Recent data showed a reduction in ATP‐binding cassette (ABC) subfamily B member 10 (ABCB10) expression following acute exposure to DOX [3]. ABCB10 is localized to the inner mitochondrial membrane and is one of four mitochondria‐specific ABC transporters. While the precise substrates for ABCB10 are unknown, its subfamily is classified as multidrug resistance transporters, and thus it has been suggested to facilitate the mitochondrial efflux of chemotherapeutics [11]. However, its tissue effects are not dependent on drug transport as ABCB10 was shown to positively regulate mitochondrial ROS production and heme biosynthesis in response to cardiac ischemia–reperfusion injury [12]. Therefore, targeting ABCB10 may have therapeutic potential for the treatment of DOX‐induced muscle weakness and fatigue.

We performed three independent experiments to test the overarching hypothesis that mitochondrial DOX retention and subsequent respiratory muscle toxicity are directly related to changes in ABCB10 expression. Utilizing a single dose of DOX and an acute timepoint, we determined whether DOX is a potential substrate for ABCB10 by measuring mitochondrial DOX concentration following overexpression and knockdown of ABCB10. In addition, we developed a clinically relevant multicycle DOX chemotherapy protocol to determine whether ABCB10 overexpression has therapeutic potential as a countermeasure to prevent respiratory muscle toxicity. These studies support the idea that DOX‐induced respiratory muscle dysfunction is associated with the mitochondrial accumulation of DOX and also demonstrate that ABCB10 overexpression can prevent DOX diaphragm toxicity. However, the effects of ABCB10 were not related to changes in DOX mitochondrial localization and retention, but rather to the maintenance of redox balance and iron homeostasis.

## 2 Methods

### 2.1 Experimental Animals

Female Sprague–Dawley rats obtained from Charles River Laboratories (Charleston, SC) were used for these experiments (Experiments 1 and 2 *n* = 10/group, Experiment 3 *n* = 8/group). Animals were housed at the University of Florida Animal Care Services Center according to guidelines set forth by the Institutional Animal Care and Use Committee (IACUC). Animals were maintained on a 12:12‐h light:dark cycle and provided food and water ad libitum. These experiments were performed in accordance with the Guidelines for the Care and Use of Laboratory Animals. The University of Florida IACUC approved these experiments.

### 2.2 Experimental Procedures

#### 2.2.1 Experiment 1

Experiment 1 tested the hypothesis that increased diaphragm expression of ABCB10 reduces the accumulation of DOX. ABCB10 expression was increased in the diaphragm via systemic delivery of an adeno‐associated virus (AAV) vector containing a muscle‐specific promoter, the gene of interest and a GFP tag (rAAV9‐MHCK7‐ABCB10, Vector Biolabs, Malvern, PA). 10^11^vg of the ABCB10 vector was diluted to a volume of 200 μL in sterile saline and administered via tail vein (ABCB10^+^). An equal volume of saline containing no vector was administered in control rats (SAL). Four weeks following administration, rats received an intraperitoneal (IP) injection of either DOX hydrochloride (20 mg/kg; TEVA pharmaceuticals, Parsippany, NJ) (DOX) or an equal volume of saline (SAL). Two days following DOX/SALINE treatment, rats underwent plethysmography, were euthanized and their diaphragms were removed and used for functional and biochemical analysis.

#### 2.2.2 Experiment 2

Experiment 2 tested the hypothesis that reduced diaphragm expression of ABCB10 increases the accumulation of DOX. ABCB10 expression was reduced in the diaphragm via systemic administration of an antisense oligonucleotide (ASO) targeting ABCB10 (Integrated DNA Technologies, Coralville, IA). The ASO was diluted in sterile saline, and 60 mg/kg was administered IP for five consecutive days prior to euthanasia (ABCB10^−^). Equal volumes of saline containing no ASO were administered in control rats (SAL). On Day 4, rats received an IP injection of either DOX (20 mg/kg) or SAL (equal volume). Two days following DOX/SAL treatment, rats underwent plethysmography, were euthanized and their diaphragms were removed and used for functional and biochemical analysis. The single dose and 2‐day timepoint were used in Experiments 1 and 2 because DOX has an elimination half‐life of 20–48 h, and our previous work verified that the cytosolic and mitochondrial concentration of DOX can be measured in the diaphragm with these conditions [3].

#### 2.2.3 Experiment 3

To test the hypothesis that increased diaphragm expression of ABCB10 protects against DOX respiratory dysfunction, rats were administered 10^11^vg of rAAV9‐MHCK7‐ABCB10 diluted to a volume of 200 μL in sterile saline or an equal volume of saline via tail vein administration. One week following ABCB10 vector (ABCB10^+^) or saline (SAL) administration, DOX (5.7 mg/kg) or SAL (equal volume) was delivered via IP injection once every 3 weeks for a total of three treatment cycles. This dose of DOX was allometrically scaled to reflect a human equivalent prescription clinically used in breast cancer treatment and corresponds to 40 mg/m^2^ [13]. Two days following the final cycle, rats underwent plethysmography, were euthanized and their diaphragms were removed and used for functional and biochemical analysis.

### 2.3 Experimental Measures

#### 2.3.1 In Vitro Diaphragm Contractile and Fatigue Properties

A strip of diaphragm muscle was dissected from the mid‐costal region of the diaphragm and suspended vertically between platinum wire electrodes with one end connected to an isometric force transducer within a jacketed tissue bath containing Krebs–Henseleit buffer at 25°C (Aurora Scientific, Aurora, ON, Canada). The muscle strip was stimulated along its entire length using supramaximal (~150%) stimulation voltage to determine the optimal contractile length (Lo). To measure the force‐frequency response, each strip was stimulated with supramaximal stimulations at optimal length with 120‐V pulses at 15–160 Hz. After a 5‐min rest, diaphragm muscle fatigue was measured by tetanic contractions using a stimulus train of 30 Hz every 2 s for 600 s with a train duration of 250 ms. Diaphragm force was normalized to cross‐sectional area to determine muscle‐specific force [14].

#### 2.3.2 Diaphragm Cross‐Sectional Area

A section of mid‐costal diaphragm was placed in optimal cutting temperature compound and frozen in liquid nitrogen‐cooled isopentane. Diaphragm muscle cross‐sections (10 μm) were cut with a cryostat (CM3050S Cryostat, Leica Biosystems, Wetzlar, Germany) and stained for α‐laminin (Sigma‐Aldrich, St. Louis, MO), myosin heavy chain I (Iowa Developmental Studies Hybridoma Bank, Iowa City, IA) and myosin heavy chain IIA (Iowa Developmental Studies Hybridoma Bank) for analysis of diaphragm muscle fibre type and cross‐sectional area. Fluorescent images were acquired using a Revolve microscope (ECHO, Laboratories, San Diego, CA, USA) and analysed with ImageJ software.

#### 2.3.3 Whole‐Body Plethysmography

Rats were placed into a plexiglass plethysmography chamber while breathing room air (21% O_2_, balanced N_2_) to acclimate and determine resting ventilation [15]. A sequential protocol was then initiated consisting of 5 min of hypercapnia (7% CO_2_, 21% O_2_, balanced N_2_) and 5 min of hypoxia (10% O_2_, balanced N_2_), with 10 min of room air breathing between each gas challenge. The pressure calibration signal, plethysmograph temperature, rat body temperature, ambient and chamber pressures and body mass were used to calculate breath‐by‐breath breathing frequency (breaths/min), tidal volume (TV; mL/breath) and minute ventilation (MV; mL/min).

#### 2.3.4 Mitochondrial Isolation

Mitochondria were isolated from the left hemidiaphragm. Diaphragm muscle was minced on ice for 5 min in 10× mg‐volume stable buffer (solution 1: 100‐mM KCl, 50‐mM MOPS, 5‐mM MgSO_4_, 1‐mM EGTA, 1‐mM ATP, 0.2% BSA, pH 7.4 at 4°C). Contents were briefly processed with a mechanical homogenizer, followed by trypsin digestion (1× mg‐volume [5‐mg trypsin/g of tissue] prepared in solution 1). Digestion was terminated with the addition of 10× mg‐volume of solution 1. Mitochondria underwent several washing steps with solution 1 and centrifugation (3500 × *g*, 10 min, 4°C), with a final pelleting spin in solution 2 (solution 1 without BSA). The mitochondrial pellet was resuspended in 250 μL of resuspension buffer (220‐mM mannitol, 70‐mM sucrose, 10‐mM Tris‐HCl and 1‐mM EGTA, pH 7.4 at 4°C).

#### 2.3.5 DOX and Doxorubicinol (DOXol) Concentration

The concentration of DOX and DOXol was determined in the mitochondrial and cytosolic fractions isolated from the diaphragms of DOX‐treated rats (*n* = 6/group). High performance liquid chromatography/electrospray ionization tandem mass spectrometry (HPLC‐ESI‐MS/MS) was performed at the University of Florida Mass Spectrometry Core as previously described [16].

#### 2.3.6 Mitochondrial Respiration

Mitochondrial respiration was measured via polarography. Twenty microlitres of isolated diaphragm mitochondria was suspended in 955 μL of buffer containing 50‐mM K‐MES, 30‐mM KCl, 10‐mM K_2_HPO_4_, 1‐mM EGTA, 5‐mM MgCl_2_‐6H_2_O, 0.005‐mM glutamate, 0.002‐mM malate and 0.05% BSA, pH 7.1 with 20‐mM creatine maintained at 37°C within an oxygraph chamber (Hansatech Instruments, Norfolk, UK). Mitochondrial respiration was performed in duplicate. Maximal ADP‐stimulated respiration (State 3) was obtained using complex I substrates (5‐mM pyruvate and 5‐mM malate) in the presence of 0.25‐mM ADP, and State 4 respiration was recorded following the complete phosphorylation of ADP. The respiratory control ratio (RCR) was then calculated by dividing State 3 by State 4 respiration. States 3 and 4 values were normalized post hoc to protein content by the Bradford method.

#### 2.3.7 Mitochondrial ROS

Hydrogen peroxide formation was measured in isolated diaphragm mitochondria using Amplex Red (Molecular Probes, Eugene, OR, USA). State 4 ROS was evaluated with 5× Wanders buffer (50‐mM MgCL_2_, 500‐mM KCl, 250‐mM MOPS, 5‐mM EGTA, 1% BSA [free fatty acid]; pH 7.1), 1‐M KH_2_PO_4_, 1 μL of Amplex Red stock, SOD stock solution, succinate and 20 μL mitochondrial resuspension. Readings were performed in a black 96‐well plate (Corning, Kennebunk, ME) with excitation at 544 nm and emission at 590 nm. Mitochondria samples were assayed in duplicate with values averaged and normalized post hoc to protein content by the Bradford method.

#### 2.3.8 Real‐Time Polymerase Chain Reaction

Total RNA was isolated from diaphragm muscle with TRIzol reagent (Life Technologies, Carlsbad, CA). RNA concentrations were determined spectrophotometrically. Superscript III First‐Strand Synthesis System for RT‐PCR (Life Technologies), using oligo(dT)20 primers, was used to reverse transcribe 3 μg of RNA. One microlitre of cDNA was added to a 25 μL PCR reaction for real‐time PCR using Taqman chemistry. Samples were run in duplicate and relative quantification of gene expression was performed using the comparative computed tomography method. ABCB10, ABCB7, ferrochelatase (Fech), mitoferrin 1 (Mfrn1), mitoferrin 2 (Mfrn2), 5′‐aminolevulinate synthase (ALAS) ALAS1, ALAS2, coproporphyrinogen oxidase (CPOX) and protoporphyrinogen (PPOX) mRNA transcripts were assayed using predesigned rat primer and probe sequences commercially available from Applied Biosystems.

#### 2.3.9 Mitochondrial Iron

Diaphragm mitochondrial iron levels were determined using a colorimetric assay kit according to the manufacturer's instructions (Sigma‐Aldrich). Samples were tested directly to measure ferrous iron and reduced to measure total iron. Ferric iron levels were derived by subtracting ferrous iron from total iron.

#### 2.3.10 Western Blot

Diaphragm muscle was homogenized in lysis buffer with protease inhibitor cocktail (Sigma‐Aldrich). Supernatant was separated from the pellet, and the Bradford method was used to assess protein content. Proteins were separated via 4%–20% precast gels (Bio‐Rad Laboratories, Hercules, CA) and transferred to nitrocellulose membranes. Immediately following transfer, membranes were incubated in Revert 700 Total Protein Stain (LI‐COR, Lincoln, NE) and rinsed twice with Revert 700 Wash Solution (LI‐COR) prior to imaging. Total protein staining was used for all western blot normalization. Stain was removed with Revert Destaining Solution (LI‐COR), and membranes were blocked with 5% nonfat milk and probed with primary antibodies diluted in Odyssey blocking buffer (LI‐COR) for the following proteins of interest: nuclear receptor coactivator 4 (NCOA4) (Abcam, Boston, MA), ferritin (Proteintech, Rosemont, IL), ATG12‐5 conjugation (Cell Signalling, Danvers, MA), acyl‐CoA synthetase long‐chain family member 4 (ACSL4) (Invitrogen, Waltham, MA) and glutathione peroxidase 4 (GPX4) (Abcam). Following overnight incubation with primary antibodies at 4°C, membranes were washed with PBS with 0.1% Tween and then incubated with corresponding IRDye secondary antibodies (LI‐COR). Membranes were imaged on an Odyssey CLx (LI‐COR), and Image Studio software (LI‐COR) was used to analyse images.

### 2.4 Statistical Analysis

Data are presented as mean ± standard error of the mean. A Shapiro–Wilk test was used to test for normal distribution of data. Comparisons between two groups were evaluated using a Student's *t*‐test or a Mann–Whitney test for nonparametric data. A two‐way analysis of variance (ANOVA) was conducted to determine whether main effects of SAL/ABCB10 or SAL/DOX treatment or a DOX × ABCB10 interaction existed. A Tukey multiple comparisons test was performed when a significant interaction was detected. Results from each two‐way ANOVA are presented on the graphs. Significance was established at *p* < 0.05.

## 3 Results

### 3.1 Experiment 1

#### 3.1.1 ABCB10 Overexpression Prevents Acute DOX‐Induced Diaphragm Weakness and Respiratory Dysfunction

**FIGURE 1:**
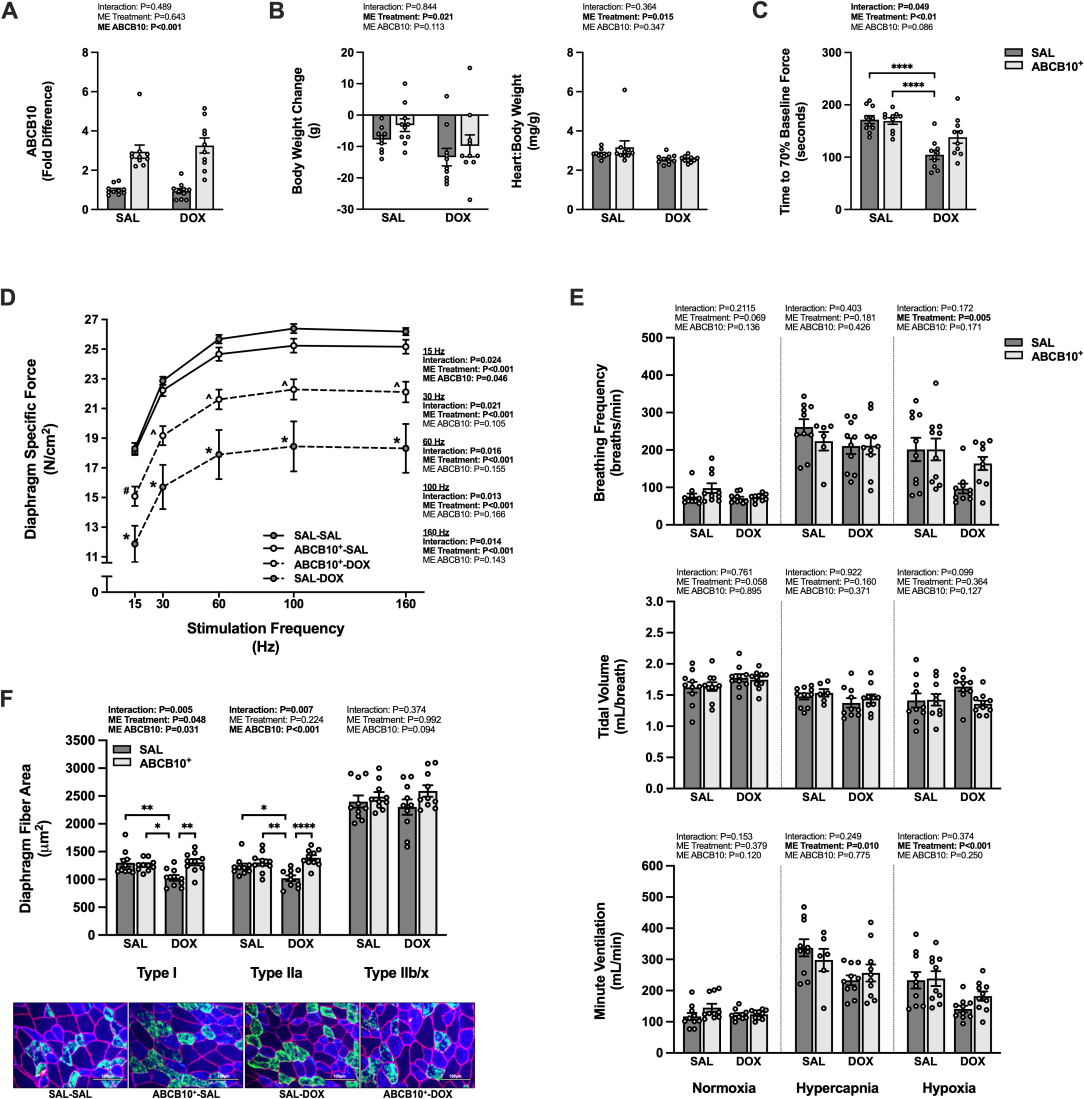
Effects of acute DOX administration and ABCB10 overexpression on markers of respiratory muscle function. (A) Diaphragm gene expression of ABCB10. (B) Body weight change from the time of DOX treatment to experimental endpoint (left) and the heart to body weight ratio (right). (C) Time to 70% of baseline diaphragm force production. (D) Diaphragm force‐frequency response. (E) Ventilatory response to normoxia, hypercapnia and hypoxia; breathing frequency (top), tidal volume (middle) and minute ventilation (bottom). (F) Diaphragm muscle cross‐sectional area and representative images. Scale bars = 100 μm. Open circles depict individual data points. Data are presented as mean ± SEM. Results from each two‐way ANOVA are shown on the graph. * = *p* < 0.05, ** = *p* < 0.01, **** = *p* < 0.0001. Force‐frequency response: * = different vs. all groups, # = different vs. SAL‐SAL and ABCB10^+^‐SAL, ^ = different vs. ABCB10^+^‐SAL; *p* < 0.05.

Diaphragm ABCB10^+^ had no effect on body weight at any experimental timepoint, but there was a main effect of DOX to induce a greater change in body weight from the time of DOX/SAL treatment to the endpoint, and there was a main effect for DOX treatment to reduce the heart:body weight ratio (Figure 1A,B). With repeated contractions, the diaphragm of the SAL‐DOX rats fatigued to 70% of baseline force more rapidly than that of the SAL or ABCB10^+^ control rats (SAL‐SAL and ABCB10^+^‐SAL, respectively) (Figure 1C). Specific force production was diminished in the SAL‐DOX group for all frequencies (15–160 Hz) compared to all other groups (Figure 1D) but was also reduced in the ABCB10^+^‐DOX group compared to both control groups at 15 Hz and compared to SAL‐SAL from 30 to 160 Hz. There was a main effect of DOX to reduce minute ventilation during a hypercapnic or hypoxic respiratory challenge (Figure 1E). The response to hypoxia occurred as a result of reduced breathing frequency. Acute DOX exposure resulted in significant atrophy of the types I and IIa fibres compared to all groups (Figure 1F). No difference between groups was seen for type IIb/x fibres.

#### 3.1.2 ABCB10 Overexpression Does Not Affect Diaphragm DOX and DOXol Content in the Mitochondrial or Cytosolic Fractions

**FIGURE 2:**
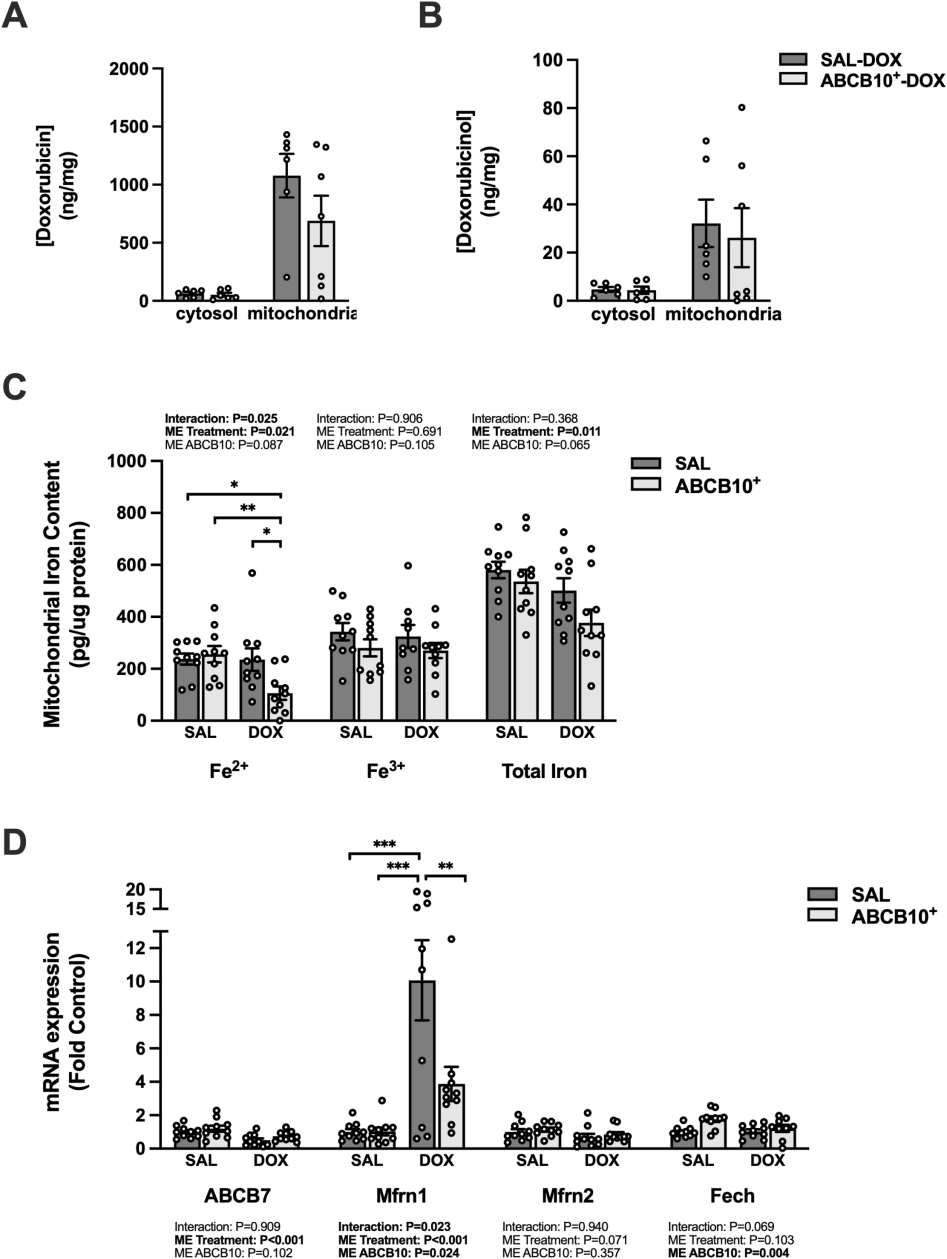
Effects of acute DOX administration and ABCB10 overexpression on markers of DOX accumulation and iron homeostasis. (A) Cytosolic and mitochondrial [doxorubicin]. (B) Cytosolic and mitochondrial [doxorubicinol]. (C) Mitochondrial Fe^2+^, Fe^3+^ and total iron content. (D) Diaphragm gene expression of ABCB7, mitoferrin1 (Mfrn1), mitoferrin2 (Mfrn2) and ferrochelatase (Fech). Open circles depict individual data points. Data are presented as mean ± SEM. Results from each two‐way ANOVA are shown on the graph. * = *p* < 0.05, ** = *p* < 0.01, *** = *p* < 0.001.

No differences were seen in the concentration of DOX or its primary metabolite DOXol in the diaphragm mitochondrial and cytosolic fractions between the SAL‐DOX and ABCB10^+^‐DOX groups (Figure 2A,B). ABCB10^+^ in DOX‐treated rats reduced the mitochondrial concentration of ferrous iron (Fe^2+^) compared to all groups; no differences between groups existed for ferric iron (Fe^3+^) and there was a main effect of DOX to reduce total iron levels (Figure 2C). There was a main effect of DOX to reduce diaphragm gene expression of ABCB7 (Figure 2D). Mfrn1 was upregulated in the SAL‐DOX group compared to all others, with no difference between groups for Mfrn2. For Fech, there was a main effect for ABCB10^+^ to increase gene expression.

### 3.2 Experiment 2

#### 3.2.1 Knockdown of Diaphragm ABCB10 Expression Does Not Exacerbate DOX‐Induced Diaphragm Weakness

**FIGURE 3:**
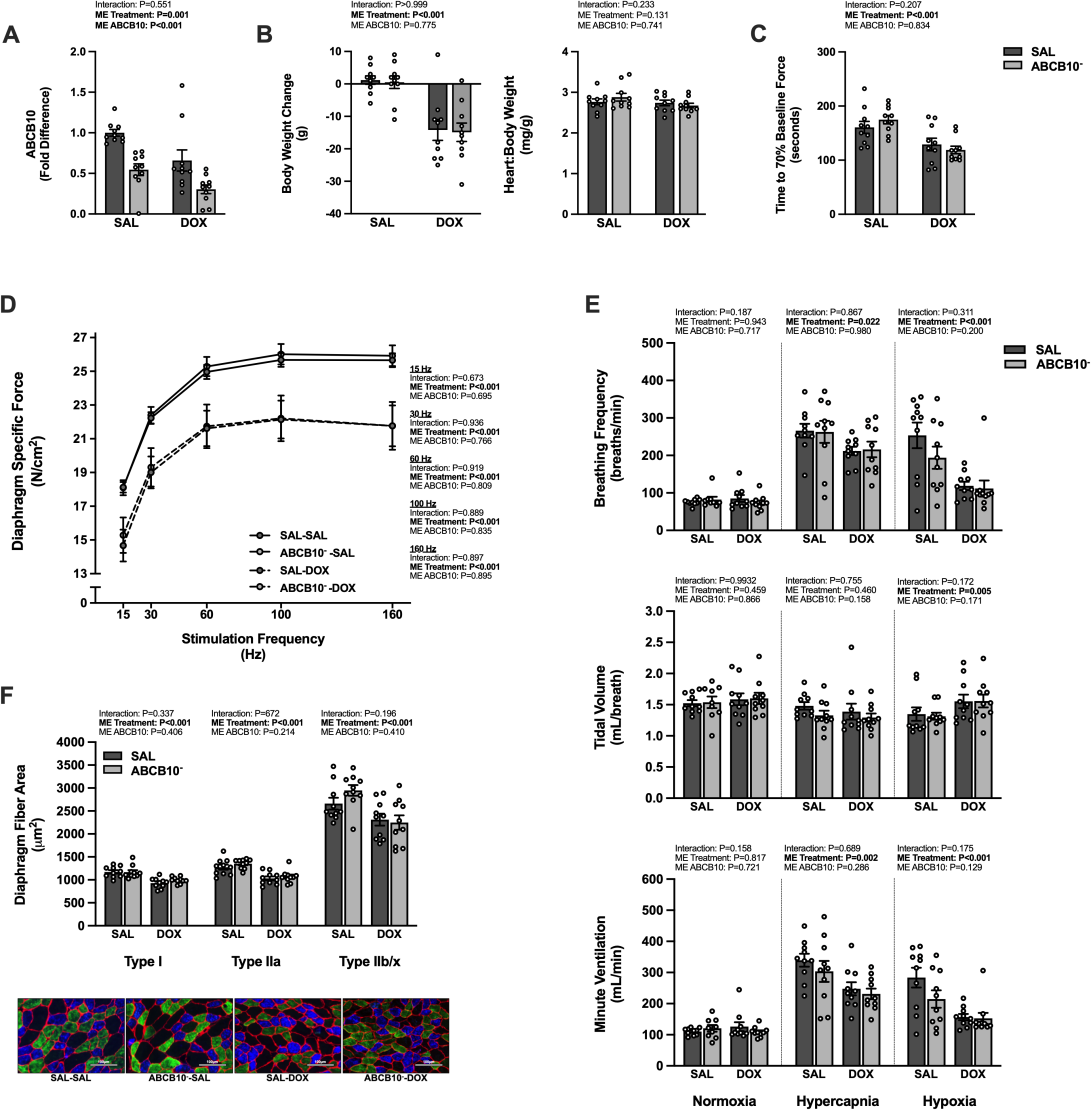
Effects of acute DOX administration and ABCB10 knockdown on markers of respiratory muscle function. (A) Diaphragm gene expression of ABCB10. (B) Body weight change from the time of DOX treatment to experimental endpoint (left) and the heart to body weight ratio (right). (C) Time to 70% of baseline diaphragm force production. (D) Diaphragm force‐frequency response. (E) Ventilatory response to normoxia, hypercapnia and hypoxia; breathing frequency (top), tidal volume (middle) and minute ventilation (bottom). (F) Diaphragm muscle cross‐sectional area and representative images. Scale bars = 100 μm. Open circles depict individual data points. Data are presented as mean ± SEM. Results from each two‐way ANOVA are shown on the graph.

Acute DOX exposure resulted in a main effect of DOX to cause body weight loss from the time of DOX/SAL treatment to the endpoint, but there was no effect of DOX or ABCB10^−^ on the heart:body weight ratio (Figure 3A,B). There was a main effect for diaphragm fatigue to occur more rapidly in the DOX‐treated groups compared to the SAL‐treated groups with no effect of ABCB10^−^ (Figure 3C). This same effect was seen for diaphragm muscle‐specific force at all frequencies tested (15–160 Hz) (Figure 3D). There was a main effect of DOX to reduce the ventilatory response to hypercapnia and hypoxia (Figure 3E). The response to hypercapnia and hypoxia occurred as a result of reduced breathing frequency. There was also a main effect of DOX to reduce the cross‐sectional area of type I, type IIa and type IIb/x diaphragm muscle fibres (Figure 3F).

#### 3.2.2 ABCB10 Knockdown Does Not Affect Diaphragm DOX and DOXol Content in the Mitochondrial or Cytosolic Fractions

**FIGURE 4:**
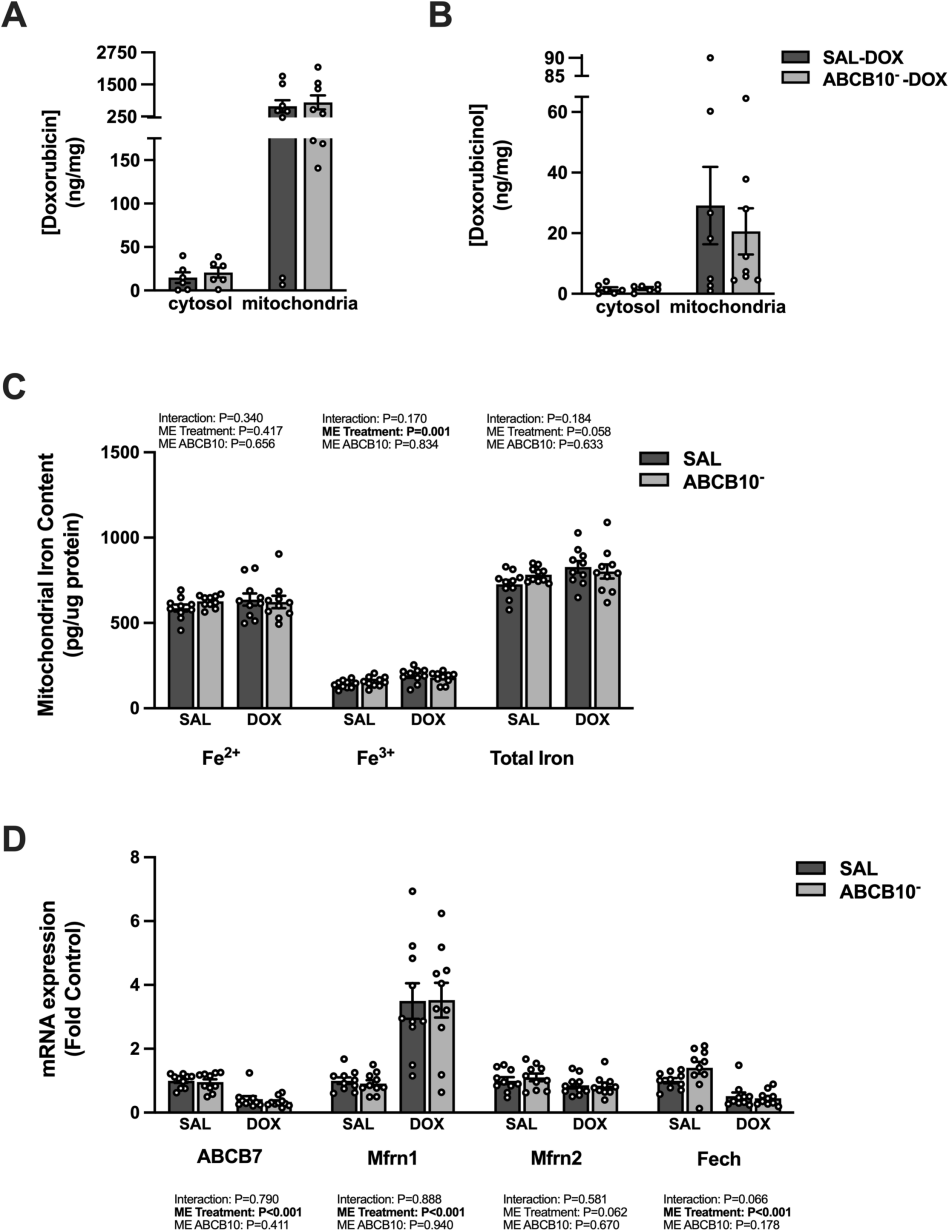
Effects of acute DOX administration and ABCB10 knockdown on markers of DOX accumulation and iron homeostasis. (A) Cytosolic and mitochondrial [doxorubicin]. (B) Cytosolic and mitochondrial [doxorubicinol]. (C) Mitochondrial Fe^2+^, Fe^3+^ and total iron content. (D) Diaphragm gene expression of ABCB7, mitoferrin1 (Mfrn1), mitoferrin2 (Mfrn2) and ferrochelatase (Fech). Open circles depict individual data points. Data are presented as mean ± SEM. Results from each two‐way ANOVA are shown on the graph.

No differences were seen in the concentration of DOX or DOXol in the diaphragm mitochondrial and cytosolic fractions between the SAL‐DOX and ABCB10^−^‐DOX groups (Figure 4A,B). There was a main effect of DOX to increase mitochondrial ferric iron content, with no difference in ferrous or total iron content (Figure 4C). A main effect of DOX to decrease ABCB7 and Fech gene expression and increase Mfrn1 was revealed (Figure 4D). No differences existed between groups for Mfrn2.

### 3.3 Experiment 3

#### 3.3.1 ABCB10 Overexpression Prevents Chronic DOX‐Induced Diaphragm Weakness and Respiratory Dysfunction

**FIGURE 5:**
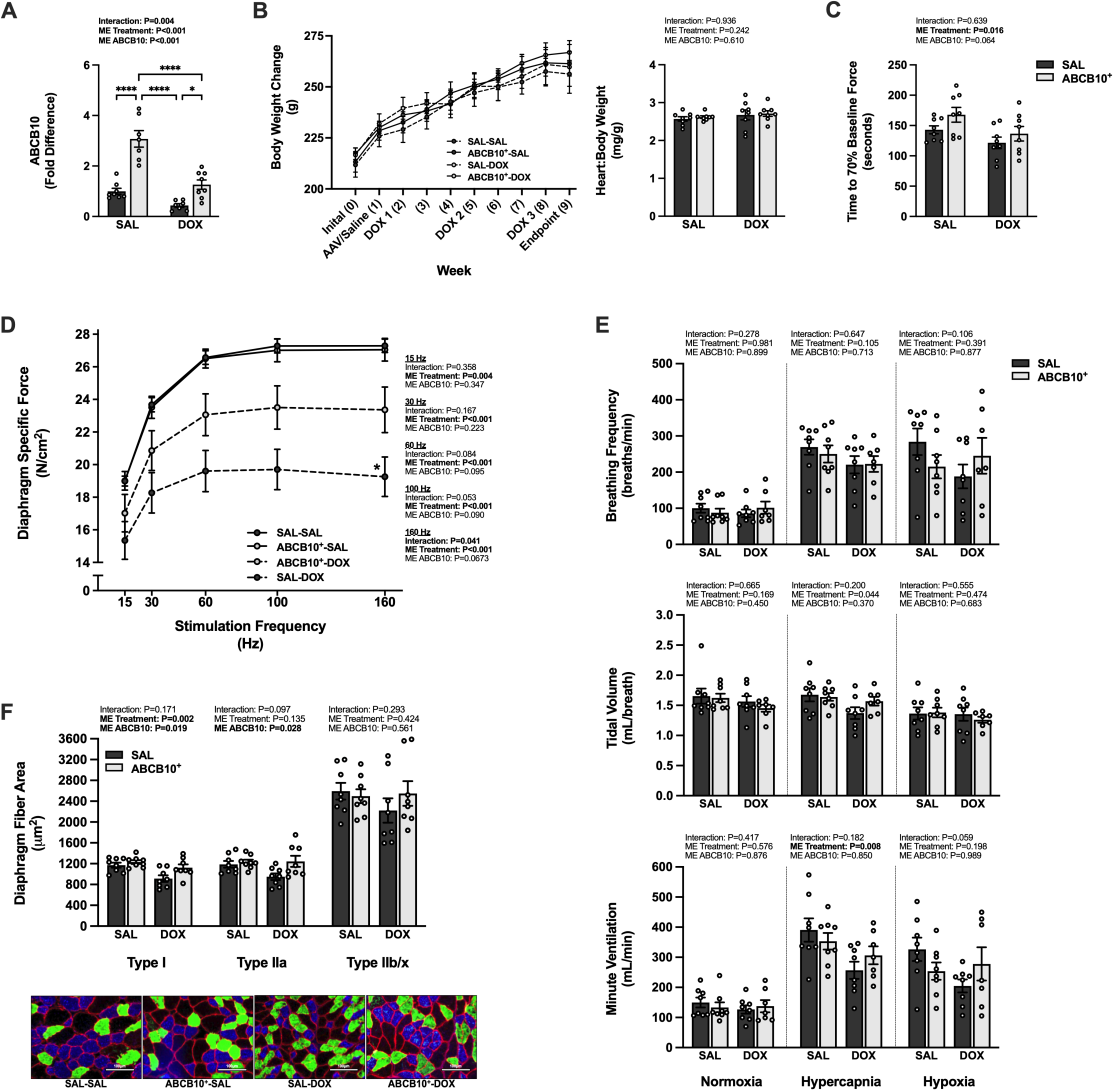
Effects of chronic DOX administration and ABCB10 overexpression on markers of respiratory muscle function. (A) Diaphragm gene expression of ABCB10. (B) Body weight change over 3 cycles of DOX (left) and the heart to body weight ratio (right). (C) Time to 70% of baseline diaphragm force production. (D) Diaphragm force‐frequency response. (E) Ventilatory response to normoxia, hypercapnia and hypoxia; breathing frequency (top), tidal volume (middle) and minute ventilation (bottom). (F) Diaphragm muscle cross‐sectional area and representative images. Scale bars = 100 μm. Open circles depict individual data points. Data are presented as mean ± SEM. Results from each two‐way ANOVA are shown on the graph. * = *p* < 0.05, **** = *p* < 0.0001. Force‐frequency response: * = different vs. all groups.

No differences in body weight were seen in response to ABCB10^+^ or repeat dosing of DOX, and there was no effect of DOX or ABCB10^+^ on the heart:body weight ratio (Figure 5A,B). Diaphragm rate of fatigue showed a main effect of DOX to reduce the time to 70% baseline force (Figure 5C). There was also a main effect of DOX to reduce diaphragm muscle specific force production at all frequencies tested (Figure 5D). At 160‐Hz force production was significantly reduced in the SAL‐DOX group compared to all other groups. There was a main effect for DOX to reduce minute ventilation during a hypercapnic respiratory challenge (Figure 5E). Repeated DOX exposure resulted in a main effect of DOX to reduce type I muscle fibre CSA, but also a main effect of ABCB10^+^ to increase type I and type IIa diaphragm muscle fibre CSA (Figure 5F).

#### 3.3.2 ABCB10 Overexpression Prevents Chronic DOX‐Induced Mitochondrial Dysfunction and Improves Iron Handling

**FIGURE 6:**
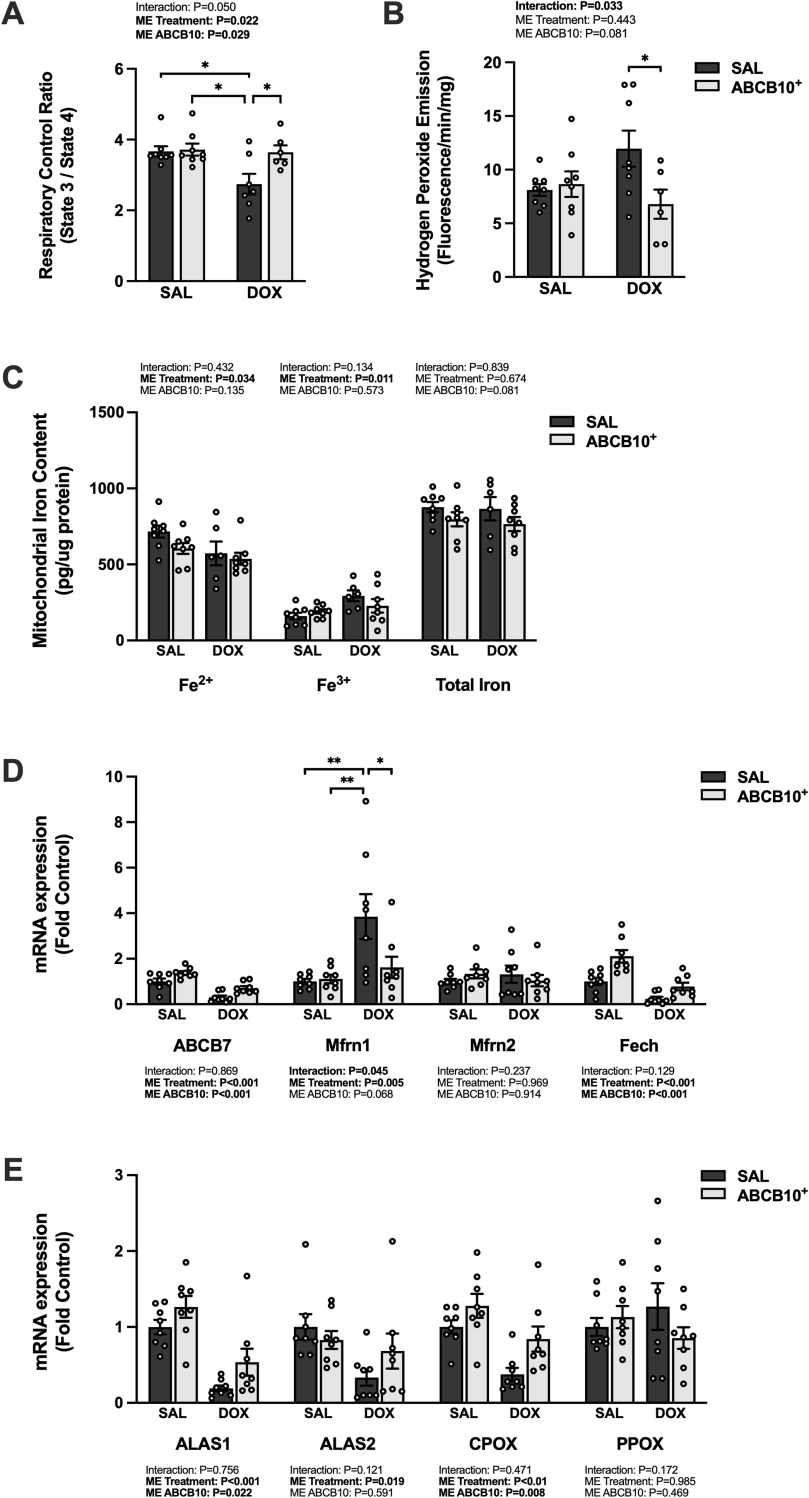
Effects of chronic DOX administration and ABCB10 overexpression on markers of DOX accumulation and iron homeostasis. (A) Mitochondrial respiratory control ratio. (B) Mitochondrial hydrogen peroxide production. (C) Mitochondrial Fe^2+^, Fe^3+^ and total iron content. (D) Diaphragm gene expression of ABCB7, mitoferrin1 (Mfrn1), mitoferrin2 (Mfrn2) and ferrochelatase (Fech). (E) Diaphragm gene expression of 5′‐aminolevulinate synthase (ALAS) ALAS1, ALAS2, coproporphyrinogen oxidase (CPOX) and protoporphyrinogen (PPOX). Open circles depict individual data points. Data are presented as mean ± SEM. Results from each two‐way ANOVA are shown on the graph. * = *p* < 0.05, ** = *p* < 0.01.

**FIGURE 7:**
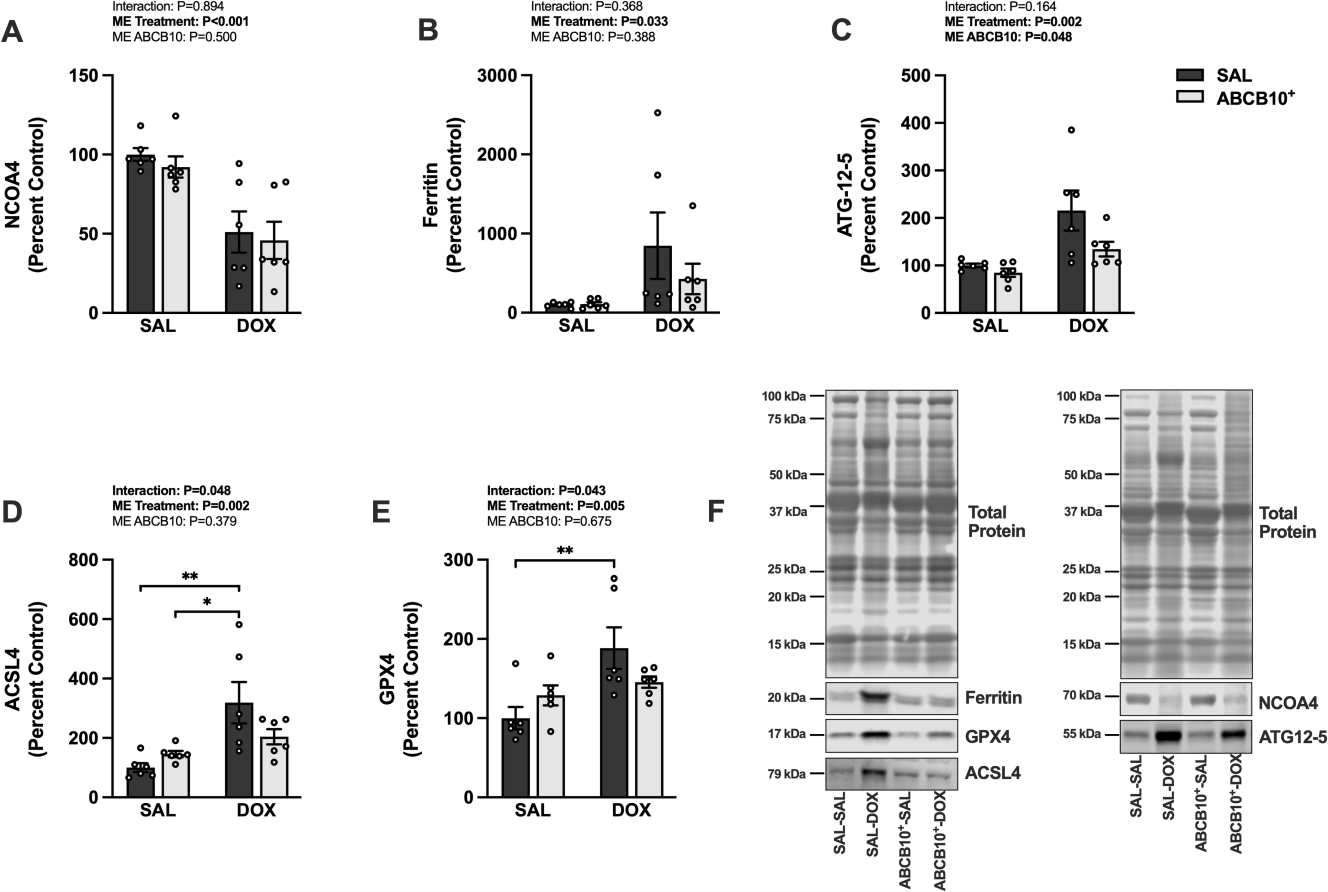
Effects of chronic DOX administration and ABCB10 overexpression on markers of ferroptosis. Diaphragm protein expression of (A) nuclear receptor coactivator 4 (NCOA4), (B) ferritin, (C) ATG12‐5 conjugation, (D) acyl‐CoA synthetase long‐chain family member 4 (ACSL4), (E) glutathione peroxidase 4 (GPX4) and (F) representative western blot images. Open circles depict individual data points. Data are presented as mean ± SEM. Results from each two‐way ANOVA are shown on the graph. * = *p* < 0.05, ** = *p* < 0.01.

Diaphragm mitochondrial RCR was decreased in SAL‐DOX rats compared to all groups and ROS production was increased compared to ABCB10^+^‐DOX (Figure 6A,B). There was a main effect of DOX to decrease mitochondrial ferrous iron and increase ferric iron.

(Figure 6C). There was a main effect of DOX to reduce ABCB7 and Fech and a main effect of ABCB10^+^ to increase ABCB7 and Fech gene expression (Figure 6C). Mfrn1 expression was increased in the SAL‐DOX group compared to all groups. There was also a main effect of DOX to reduce ALAS2 and CPOX and a main effect of ABCB10^+^ to increase their gene expression (Figure 6E). ALAS2 was downregulated in the SAL‐DOX group compared to SAL‐SAL. Diaphragm protein expression of NCOA4 showed a main effect of DOX to reduce its levels (Figure 7A). There was also a main effect of DOX to increase ferritin protein expression (Figure 7B). The ATG12‐5 conjugation product showed a main effect of DOX to increase its level and also a main effect of ABCB10^+^ to decrease it (Figure 7C). Protein expression of ACSL4 was increased in the SAL‐DOX group compared to both SAL‐treated control groups (Figure 7D) and GPX4 was also increased in the SAL‐DOX group compared to SAL‐SAL (Figure 7E).

## 4 Discussion

DOX is recognized as one of the most powerful cancer chemotherapeutics; however, its use is greatly limited due to toxicity to noncancerous cells [17]. Off‐target distribution of DOX to healthy cells is highly correlated to tissue impairment, and our group has demonstrated that DOX accumulation in the diaphragm muscle is associated with respiratory weakness and fatigue [3]. Respiratory muscle dysfunction is established in cancer patients and is a leading cause of dyspnea, exercise intolerance and sedentary behaviour [18, 19]. These symptoms adversely affect the ability to perform activities of daily living and quality of life, which are important determinants of survival [8, 20]. Evidence indicates that diaphragm dysfunction results from the rapid influx of DOX to the mitochondrial matrix, resulting in futile redox cycling and free radical formation, causing cellular oxidation and protein breakdown [21, 22]. The underlying mechanisms required for this signalling cascade remain unclear. Thus, no clinically approved therapeutic countermeasures exist to combat this important problem. Our data suggest that increased ABCB10 expression is sufficient to prevent DOX‐induced diaphragm muscle atrophy and weakness, but it is not required to maintain respiratory function, as our results show that reducing its expression had no significant effect on either saline‐ or DOX‐treated rats. A detailed description of our findings follows.

### 4.1 ABCB10 Overexpression Prevents DOX‐Induced Respiratory Muscle Dysfunction and Fatigue

ABCB10 is a mitochondria‐localized ABC transporter demonstrated to play a role in cellular protection from oxidative stress and regulation of heme and iron metabolism [23]. Previous work from our group postulated that ABCB10 expression is associated with diaphragm function following DOX administration, as exposure to a single 20 mg/kg dose of DOX for 48 h significantly decreased specific force production, cross‐sectional area and ventilatory function [3]. Concomitant with these detriments was the accumulation of DOX predominantly within the muscle mitochondrial fraction, mitochondrial ROS production, mitochondrial dysfunction and a reduction in diaphragm ABCB10 expression. Interestingly, when ABCB10 levels were preserved via preconditioning exercise, many of these adverse effects were alleviated [3]. Our data demonstrate that, independent of exercise training, diaphragm overexpression of ABCB10 is sufficient to prevent DOX respiratory dysfunction. Increased ABCB10 attenuated the decline in specific force production, muscle fibre size and ventilation associated with acute DOX exposure. In addition, increasing diaphragm expression of ABCB10 was also effective at preventing diaphragm dysfunction associated with a DOX treatment protocol designed to mimic standard DOX dosing typically prescribed to breast cancer patients (i.e., multiple cycles administered once every 3 weeks, ~40 mg/m^2^/cycle). Thus, therapeutics targeting ABCB10 to increase its expression may have the potential to protect against DOX‐induced respiratory muscle dysfunction and fatigue.

### 4.2 Reduction in ABCB10 Expression Does Not Cause Diaphragm Dysfunction

Investigation into the pathophysiology of DOX respiratory toxicity and ABCB10 expression revealed that while increasing diaphragm expression of ABCB10 can prevent DOX‐induced respiratory muscle dysfunction, decreasing ABCB10 does not cause diaphragm dysfunction and atrophy. Specifically, in both saline‐ and DOX‐treated rats, an ~50% reduction in ABCB10 gene expression had no effect on diaphragm function compared to treatment‐matched controls. This reduction in diaphragm ABCB10 in the healthy control rats to expression levels comparable to the SAL‐DOX treated group is significant because it suggests that the DOX‐induced decrease in ABCB10 is not responsible for the acute respiratory muscle deficits caused by DOX treatment. These data suggest that ABCB10 is not required to maintain normal respiratory muscle function, and its reduced expression does not exacerbate DOX diaphragm toxicity. This finding in healthy, saline‐treated rats parallels findings in the hearts of ABCB10^+/−^ mice [12]. These mice display an ~50% reduction in ABCB10 expression, but no difference in cardiac function during standard conditions compared to wild‐type mice. Differences between our data in skeletal muscle and previous findings in cardiac muscle emerge when ABCB10^+/−^ mice are subjected to cardiac stress, with diastolic pressures increased and systolic pressures decreased in response to ischemia–reperfusion [12]. In addition, cardiomyocyte‐specific deletion of ABCB10 revealed a reduced lifespan of ~12 months in these mice [24]. Thus, tissue‐specific differences in the requirement for ABCB10 may exist. There is also the potential for the DOX‐induced reduction in ABCB10 to mask the effects of the ASO treatment, as the increase in oxidative stress and reduction in heme synthesis associated with ABCB10 knockdown in cardiomyocytes is already present following DOX treatment [25].

### 4.3 ABCB10 Overexpression Prevents DOX‐Induced Mitochondrial Dysfunction and Improves Iron Regulation

The concentration gradient, multidrug resistance protein expression and presence of DOX‐binding structures facilitate the rapid accumulation of DOX within muscle tissue [26]. Upon entry, DOX preferentially localizes to the mitochondria due to its affinity for the phospholipid cardiolipin on the inner mitochondrial membrane [27]. In the mitochondria, DOX increases superoxide production via redox cycling at complex I. Specifically, DOX can be reduced by NADPH to form a semiquinone radical. In the presence of oxygen, the DOX semiquinone is oxidized, generating superoxide and reforming the DOX molecule [28]. In the absence of oxygen, the DOX semiquinone can be converted into its highly toxic primary metabolite DOXol. DOX also promotes ROS production in the mitochondria via formation of a DOX‐Fe complex. This complex can cycle iron between its Fe^2+^ and Fe^3+^ forms, resulting in free radical formation [29]. The resultant increase in oxidative damage severely impairs mitochondrial function and stimulates cell death.

Mitochondrial and muscle dysfunction develop proportional to the tissue levels of DOX [3, 30]. Following cellular and mitochondrial entry, DOX retention is presumably related to drug efflux protein expression [11]. The DOX exporters located in the cellular membrane are well established. However, the proteins required to remove DOX from the mitochondria are unclear. While conflicting reports exist [31, 32], in a mouse model of hereditary hemochromatosis, changes in cardiac ABCB8 expression were reported to regulate DOX retention and cardiotoxicity, with decreased ABCB8 corresponding to DOX retention and ABCB8 overexpression corresponding with DOX efflux [31]. Based on its structure, location and classification in the ABC transporter B subfamily, ABCB10 was also hypothesized to mediate drug transport from the matrix to the inner mitochondrial membrane [11]. Our data do not support the postulate that DOX is a substrate for ABCB10, as neither overexpression nor knockdown of DOX had an impact on the cytosolic and mitochondrial concentration of DOX or its primary metabolite DOXol.

Alternatively, ABCB10 may provide protection against DOX respiratory toxicity by affecting mitochondrial redox balance and iron homeostasis [33]. In addition to its homodimer configuration, ABCB10 also forms an oligomeric complex with Mfrn1 and Fech. ABCB10 is thought to stabilize the expression and activity of these proteins to regulate iron import and protect against mitochondrial iron‐induced oxidative damage [34]. Mfrn1 imports Fe^2+^ into the mitochondrial matrix, where it is used to generate heme and iron–sulfur clusters. DOX impairs electron transport chain efficiency and diminished skeletal muscle force production is attendant with damage to heme and iron–sulfur cluster containing respiratory chain complexes [35]. Disruption to heme and iron–sulfur cluster biosynthesis induces iron overload in the mitochondria, increasing DOX‐Fe binding, free radical production, lipid peroxidation and ferroptosis [36]. Evidence of impaired iron handling was present in the rats receiving 3 cycles of DOX, with mitochondrial Fe^3+^ iron content increased in this group. Gene expression of Mfrn1 was upregulated, indicating a capacity for increased iron uptake into the matrix; however, markers of heme synthesis (i.e., ALAS1/2, CPOX and Fech) were all decreased. These DOX‐induced shifts in signalling were accompanied by reduced mitochondrial function and increased ROS emission. In addition, the increase in ACSL4 and GPX4 in the DOX‐treated group also indicates impaired iron handling and is markers of lipid peroxidation, oxidative damage and diaphragm sensitivity to ferroptosis [37]. Our data support a role for ABCB10 in the regulation of diaphragm mitochondrial iron homeostasis, as mitochondrial Fe^2+^ iron content was reduced, mitochondrial function was preserved and each gene marker of iron import and heme synthesis was returned to control levels when DOX was administered to ABCB10 overexpressing rats.

Finally, while the single, high‐dose DOX administration model is well‐suited to assess DOX clearance from tissue due to its short elimination half‐life of up to 48 h, it is important to note that DOX is known to cause both cardiac and diaphragm toxicity [3, 16, 38]. Thus, while the primary objective of this study was to further understand the effects of DOX on impaired respiratory muscle function, our results are limited by the absence of cardiac functional and biochemical assessment. Previous work by our group demonstrated a tissue‐specific difference in ABCB10 gene expression following DOX treatment, with DOX eliciting no change in ABCB10 expression in the heart but a decrease in the diaphragm, suggesting differential effects of altered ABCB10 expression between the two muscles [3]. Further mechanistic insight into ABCB10 and its downstream effects is needed. In addition, both cardiac and skeletal muscle dysfunction are hallmarks of DOX chemotherapy; however, the relationship between these effects is unknown. Evidence supports the fact that cardiomyopathy promotes respiratory muscle impairment, but data have also shown that respiratory weakness can increase cardiovascular disease risk [39, 40]. Thus, additional studies are needed to determine the precise relationship between DOX cardiac and respiratory muscle dysfunction and whether targeting ABCB10 expression has the potential to target both conditions.

## 5 Conclusions

Clinical examination of respiratory function in cancer patients receiving DOX chemotherapy treatment has revealed significant reductions in respiratory muscle strength and greater chronic activity‐related dyspnea, likely due to diaphragm muscle weakness [4, 5, 6]. Currently, the mechanisms responsible for these deleterious effects are unclear and no therapeutic countermeasure exists. Work in this field supports the notion that DOX accumulates rapidly within diaphragm mitochondria following administration [3]. DOX localized to the mitochondria has the potential to induce cell death through free radical production, which triggers cell death pathways. Specifically, in the heart, oxidative damage as a result of iron overload and impaired iron handling is a primary mechanism underlying DOX cardiotoxicity [32, 36]. Our data demonstrate for the first time that DOX‐induced diaphragm dysfunction also results from mitochondrial dysfunction, ROS production and disrupted heme synthesis signalling. In addition, these results suggest that ABCB10 can be used as a therapeutic target to preclude DOX respiratory toxicity by preventing iron dysregulation. Future work is needed to determine the efficacy of targeting ABCB10 or its downstream pathway in a combined DOX and tumour model.

## Funding

This work was supported by the National Institutes of Health R01 HL146443, S10 OD021758‐01A1 and S10 OD030250‐01A1.

## Conflicts of Interest

The authors declare no conflicts of interest.
